# Evaluating the Anti-Inflammatory and Antioxidant Effects of Broccoli Treated with High Hydrostatic Pressure in Cell Models

**DOI:** 10.3390/foods10010167

**Published:** 2021-01-15

**Authors:** Yi-Yuan Ke, Yuan-Tay Shyu, Sz-Jie Wu

**Affiliations:** Department of Horticulture and Landscape Architecture, National Taiwan University, No. 1, Section 4, Roosevelt Road, Taipei 10617, Taiwan; r07628208@ntu.edu.tw (Y.-Y.K.); tedshyu@ntu.edu.tw (Y.-T.S.)

**Keywords:** high hydrostatic pressure, broccoli, Isothiocyanates, sulforaphane, erucin, anti-inflammatory, antioxidant

## Abstract

Isothiocyanates (ITCs) are important functional components of cruciferous vegetables. The principal isothiocyanate molecule in broccoli is sulforaphane (SFN), followed by erucin (ERN). They are sensitive to changes in temperature, especially high temperature environments where they are prone to degradation. The present study investigates the effects of high hydrostatic pressure on isothiocyanate content, myrosinase activity, and other functional components of broccoli, and evaluates its anti-inflammatory and antioxidant effects. Broccoli samples were treated with different pressures and for varying treatment times; 15 min at 400 MPa generated the highest amounts of isothiocyanates. The content of flavonoids and vitamin C were not affected by the high-pressure processing strategy, whereas total phenolic content (TPC) exhibited an increasing tendency with increasing pressure, indicating that high-pressure processing effectively prevents the loss of the heat-sensitive components and enhances the nutritional content. The activity of myrosinase (MYR) increased after high-pressure processing, indicating that the increase in isothiocyanate content is related to the stimulation of myrosinase activity by high-pressure processing. In other key enzymes, the ascorbate peroxidase (APX) activity was unaffected by high pressure, whereas peroxidase (POD) and polyphenol oxidase (PPO) activity exhibited a 1.54-fold increase after high-pressure processing, indicating that high pressures can effectively destroy oxidases and maintain food quality. With regards to efficacy evaluation, NO production was inhibited and the expression levels of inducible nitric oxide synthase (iNOS) and Cyclooxygenase-2 (COX-2) were decreased in broccoli treated with high pressures, whereas the cell viability remained unaffected. The efficacy was more significant when the concentration of SFN was 60 mg·mL^−1^. In addition, at 10 mg·mL^−1^ SFN, the reduced/oxidized glutathione (GSH/GSSG) ratio in inflammatory macrophages increased from 5.99 to 9.41. In conclusion, high-pressure processing can increase the isothiocyanate content in broccoli, and has anti-inflammatory and anti-oxidant effects in cell-based evaluation strategies, providing a potential treatment strategy for raw materials or additives used in healthy foods.

## 1. Introduction

*Brassica oleracea* var. *italica* (broccoli) is a vegetable widely consumed worldwide. It is derived from genetic mutations and the evolution of wild cabbage, and is a cultivar of *Brassica oleracea*, which belongs to the family Brassicaceae together with cabbage, gai lan, and cauliflower. Broccoli is rich in a variety of nutrients, including vitamin A, vitamin C, dietary fibers, and isothiocyanates. Among these, isothiocyanates are formed primarily through hydrolysis of glucosinolates by the enzyme myrosinase, and are the most representative functional component in cruciferous vegetables. They can inhibit the proliferation, development, and metastasis of cancer cells, regulate the production of inflammation-related factors, and enhance the expression of antioxidant-related proteins [1,2,3,4]. The most abundant isothiocyanate molecule found in broccoli is sulforaphane, followed by erucic acid [5].

Myrosinases and glucosinolates not only comprise the isothiocyanate production system in plants but also act as a chemical defense mechanism. Under normal circumstances, the myrosinase enzyme is in an inactive state and the glucosinolates are stored in plants in a precursor form since these two are spatially separated. When attacked by herbivores, insects, or microorganisms, the cells are destroyed, releasing these enzymes and glucosinolate molecules, which then interact to produce the biologically active isothiocyanates, nitriles, and thiocyanates, such as, sulforaphanes [5,6]. The degree of myrosinase hydrolysis and the types of products formed are strongly affected by environmental changes, including the substrates and cofactors of myrosinase, the presence of specific proteins, pH, stress, carbon dioxide concentration, and temperature. When the pH of the hydrolysis environment is slightly acidic or neutral, the principal product produced are isothiocyanates, whereas under acidic conditions (pH < 3) or in the presence of iron or epithiospecifier protein (ESP), nitriles are produced instead, which, unlike the isothiocyanates, have no physiological effects [5,7,8].

The inflammatory response is the natural defense mechanism activated by the body when subjected to noxious stimuli. A proper inflammatory response not only protects the human body from injuries to tissues and microbial invasion, but also increases the ability of tissues and cells to restore stability and enhance the immune system [9,10]. During the inflammatory response processes, inflammation-related factors are activated to promote the production of inflammatory mediators (e.g., TNF-α and IL-6). Some inflammatory mediators may drive blood vessels to be remodeled during inflammation [11]. The oxidative stress leads to large amounts of reactive oxygen species (ROS) being produced [12]. In addition, cells and tissues absorb more oxygen due to swelling, leading to gradual accumulation of ROS. Inflammation and oxidative stress increase the likelihood of injury or pathology to tissues and cells, which can lead to immune-related diseases such as cancers and multiple sclerosis (neurodegenerative diseases) [13].

High-pressure processing (HPP), which is also known as high hydrostatic pressure (HHP) processing or ultra-high pressure (UHP) processing, is a non-thermal processing technology that has undergone rapid development in recent years. Its mechanism of action is primarily based on the use of liquids as a pressure transmission medium. Food processing under high-pressure environments at appropriate temperatures and times can inactivate microorganisms and enzymes, thereby achieving sterilization, prolonging shelf life, and reducing the use of chemical preservatives. When compared to traditional thermal processing strategies, this technique can do a much better job in preserving nutrients, flavor, appearance, and texture without any heat treatment [14,15,16]. Since isothiocyanates and their production systems are easily damaged in high-temperature environments, many studies have investigated this problem by using high-pressure techniques to treat cruciferous crops, and have noticed that not only was the isothiocyanate content retained, but there was also a tendency of improvement in the content [8,17,18,19]. Current research in this topic is focused on investigating cruciferous crops, such as samples of cabbage or broccoli sprouts, and the efficacy evaluation is based primarily on purified isothiocyanates. In this study, the No. 42 broccoli grown commercially in Taiwan was selected as the sample for processing under different high-pressure conditions to investigate the effects of high-pressure processing techniques on the changes in isothiocyanate content, myrosinase activity, and other functional components in broccoli, and also to develop the optimal processing conditions, to analyze the mechanisms behind these changes, and to evaluate efficacy through cell-based experiments.

## 2. Materials and Methods

### 2.1. Materials/Processing

Fresh broccoli was purchased from Erlun Produce Cooperative, Yunlin County, Taiwan. The broccoli was cleaned and was cut into 2-cm pieces under the florets, and equal amounts of broccoli was divided into several clean airtight bags and vacuum-sealed. The bags were then randomly divided into two groups: control group and high-pressure group. The high-pressure group was further subdivided into 9 batches based on the applied pressure and the incubation time, namely 200 MPa (3, 10, 15 min), 400 MPa (3, 10, 15 min), and 600 MPa (3, 10, 15 min). The high-pressure groups were placed into an HPP600 MPa/6.2 L (Bao Tou KeFa High Pressure Technology Co. Ltd., Inner Mongolia, Baotou, China) high-pressure apparatus for high-pressure treatment, and then allowed to stand at room temperature for 1 h to allow the myrosinase enzyme and glucosinolates to react with each other [18]. The broccoli samples were blanched in boiling water for 1 min to inactivate the polyphenol oxidase (PPO) and Peroxidase (POD) activities to reduce the browning reaction, to retain the highest commercial values. After cooling, the broccoli was freeze-dried and ground using a food processor. This was followed by filtration through a mesh and the powder was stored at −20 °C for use in subsequent analyses.

### 2.2. Extraction and High Performance Liquid Chromatography (HPLC) Analysis of Isothiocyanates

#### 2.2.1. Preparation of the Extract

The isothiocyanate extraction process was performed as described previously by Hwang and Lim (2014) [12]. A total of 0.5 g of broccoli powder was added to a 6 mL solution of 80% methanol (Macron Fine Chemicals, Center Valley, PA, USA) and the extraction process was performed with constant shaking at 1260 rpm for 1 h, followed by centrifugation at 10,000× *g* and 4 °C for 20 min. The supernatant was collected and the precipitate was resuspended with the same volume of 80% methanol, followed by extraction and centrifugation under the same conditions. The two supernatants were combined as a crude isothiocyanate extract and stored at −20 °C.

#### 2.2.2. HPLC

The analysis was performed as described by You et al. (2008) [20], with modifications. The analytical instrument used for this process was a Waters^TM^ 600 series Controller pump with a Waters 717 Plus Autosampler and SPD-20AV UV-VIS detector (Shimazu Co., Kyoto, Japan). A C18 reversed-phase chromatography column (Zorbax Eclipse XDB C-18, 4.6 × 150 mm) was used for the separation. The detection wavelength was set to 241 nm, and the sample injection volume was set to 20 μL. Water and methanol were used as the HPLC mobile phases. Initial conditions consisted of 10% methanol, followed by a linear increase to 90% methanol at 40 min, reduced to 10% at the end of the 50 min analysis time, and the column was then equilibrated at 10% methanol for 10 min. The mobile phase flow rate was kept at 1 mL·min^−1^.

### 2.3. Chemical Characterization

#### 2.3.1. Polyphenol Determination

The Folin–Ciocalteu reagent (Sigma Chemical Inc., St. Louis, MO, USA) was used to determine the content of the phenolic compounds in broccoli. A total of 0.1 g of broccoli powder was taken and suspended in 3 mL of an 80% methanol solution and the extraction process was performed for 1 h, followed by centrifugation of the homogenate at 10,000× *g* and 4 °C for 20 min. The supernatant was collected, and the precipitate underwent an extraction process for a second time in the same manner as described above. The two supernatants were combined and used as the crude extract. A total of 60 μL of extract and 60 μL of Folin–Ciocalteu reagent were mixed with 480 μL of water and was placed in the dark and allowed to react for 90 min. The change in absorbance was measured at a wavelength of 760 nm. The concentration of the TPC is expressed in GAE mg·g^−1^. Gallic acid (Sigma Chemical Inc., St. Louis, MO, USA) was used as a standard for plotting the standard curve [21].

#### 2.3.2. Flavonoid Determination

The sample extraction was performed, as described above, for the flavonoid content determination. The supernatants of the two extractions were combined and used as the crude extract. A total of 150 μL of the extract was dissolved in 450 μL 95% ethanol, a 30 μL 10% aluminum chloride solution (AlCl_3_) (Thermo Fisher Scientific Inc., Waltham, MA, USA), 30 μL potassium acetate (Merck KGaA, Darmstadt, DA, Germany), and 840 μL water, and allowed to react at 25 °C for 30 min. Then, the change in absorbance was measured at a wavelength of 415 nm. The standard curve was plotted using quercetin as a standard, and the concentration is expressed as QuE mg·g^−1^ [22].

#### 2.3.3. Vitamin C Determination

A total of 0.05 g broccoli powder was dissolved in 0.95 µL ddH_2_O (double-distilled H_2_O) to yield a 20-fold diluted solution. A reflectometer (Merck KGaA, Darmstadt, Germany) along with its Ascorbinsaure-test (Reflectoquant^®^116981) was used to determine the content of vitamin C in the solution. The concentration is expressed as mg·g^−1^.

### 2.4. Enzyme Activity Assays

#### 2.4.1. Myrosinase Activity

The experimental method followed is performed as described by Yuan et al. (2010) [23], Li et al. (2008) [24], and Zhao et al. (2008) [25], with slight modifications. A total of 0.05 g of broccoli powder was dissolved in 0.45 g of water, and then 1.8 mL of MES buffer (50 mM, pH 6.0) was added and the mixture was extracted with shaking for 5 min and centrifuged at 10,000× *g* and 4 °C for 10 min, and the supernatant was collected. A total of 100 μL of 1 mM glucoraphanin (USBiological Inc., Salem, MA, USA) was mixed with 20 μL of supernatant and allowed to react at 40–60 °C for 15 min. Then, 240 μL of DNS reagent (Sigma Aldrich Inc., St. Louis, MO, USA) was added and the reaction was carried out at 100 °C for 5 min, and then immediately cooled by placing it in an ice bath. After cooling to room temperature, 720 μL of water was added and mixed well, and a spectrophotometer was used to measure absorbance change at a wavelength of 540 nm. A standard curve was made using glucose solution as standard and the concentration was expressed as μmol glucose (Sigma Aldrich Inc., St. Louis, MO, USA) produced per min (μmol·g^−1^·min^−1^).

#### 2.4.2. Ascorbate Peroxidase (APX) Activity

The APX activity measurements were performed as described by Chen and Liu (2012) [26], with slight modifications. A total of 10 mL extraction solvent (containing 100 mM KH_2_PO_4_ (J.T. Baker Chemical Inc., Oklahoma, PA, USA), pH 7.8; 1% Triton X-100 (Sigma Chemical Inc., St. Louis, MO, USA); 1 mM EDTA-Na_2_ (Sigma Chemical Inc., St. Louis, MO, USA) was added to 0.5 g broccoli powder and the mixture was centrifuged at 10,000× *g* and 4 °C for 20 min. Then, the supernatant was collected, with the enzyme extract at 0.047–0.077 mg/g protein, and the protein content was determined by the Bradford method, with the standard curves prepared using BSA. A 0.5 mL KH_2_PO_4_ (250 mM, pH 7), 0.05 mL EDTA-Na_2_ (0.5 mM), 0.2 mL H_2_O_2_ (10 mM), 0.2 mL ascorbic acid (Honeywell Riedel-de Haen, Seelze, Germany), and 0.05 mL enzyme extract was sequentially added to the quartz tube, and the changes in absorbance were measured immediately after mixing at a wavelength of 290 nm within 5 min. The APX activity was calculated using the extinction coefficient of H_2_O_2_ (2.8 mM^−1^ cm^−1^) and the enzyme activity was expressed in units of mmol ascorbate min^−1^ mg^−1^ protein.

#### 2.4.3. Peroxidase (POD) and Polyphenol Oxidase (PPO) Activities

The enzymatic activities of these two enzymes were determined as described by Yang (2016) [27], with slight modifications. A total of 0.5 g of broccoli powder was added to 10 mL of a 0.2 M sodium phosphate buffer solution (pH 6.5) containing 1% PVPP (Sigma Chemical Inc., St. Louis, MO, USA), mixed well; the extraction was conducted by continuous shaking for 5 min, followed by centrifugation at 4 °C and 10,000× *g* for 20 min. The supernatant was collected and used as the enzyme extract. Peroxidase activity was determined by mixing 25 µL of the extract with 2.7 mL of a sodium phosphate buffer (pH 6.5), 200 µL of 1% p-phenylenediamine (Sigma Chemical Inc., St. Louis, MO, USA), and 100 μL of 1.5% hydrogen peroxide (Honeywell Riedel-de Haen, Seelze, Germany), measuring the absorbance at a wavelength of 485 nm every min for 10 min. Polyphenol oxidase activity was determined by mixing 100 μL of the enzyme extract and 3 mL of 0.15 M catechin (Sigma Chemical Inc., St. Louis, MO, USA), measuring the change in absorbance at a wavelength of 420 nm every min for 10 min. The activity of the two enzymes is expressed in the units of absorbance change per min (∆A·min^−1^).

### 2.5. Cell Culture

RAW264.7 mouse macrophages were purchased from the Bioresources Collection and Research Center (BCRC) of the Food Industry Research and Development Institute (Hsinchu, Taiwan). The cells were cultured in Dulbecco’s Modified Eagle Medium (DMEM) (Thermo Fisher Scientific Inc., Waltham, MA, USA), containing 10% fetal bovine serum (FBS) (Thermo Fisher Scientific Inc., Waltham, MA, USA) and NaHCO_3_ (Merck KGaA, Darmstadt, Germany), and placed in a 37 °C incubator containing 5% carbon dioxide (CO_2_) for growth.

### 2.6. Cell Viability Assay

The 3-(4,5-dimethylthiazol-2-yl)-2,5-diphenyltetrazolium bromide (MTS) assay is a method for evaluating the toxicity of target substances to cells. Cells were seeded in a 96-well plate at 1 × 10^4^ cells per well (cells·mL^−1^), allowed to grow for a day, and then were treated with LPS (2 μg·mL^−1^) and the broccoli extract and allowed to react for a further 24 h. The medium was then changed and CellTiter 96^®^ AQueous (Promega Co., Madison, WI, USA) One Solution was added, followed by the incubation of the cells for 1 h. Then, the change in absorbance was measured at a wavelength of 490 nm.

### 2.7. Measurement of Nitric Oxide (NO) Production

NO production was indirectly assessed by measuring the nitrite levels in the cultured media and serum determined by a colorimetric method based on the Griess reagent (Bio-Vision, Milpitas, CA, USA). Briefly, the cells were seeded in a 24-well plate at 1 × 10^5^ cells per well (cells·mL^−1^). After culturing for 24 h, the positive control group and the treatment group were first activated with LPS and treated with DMEM (Thermo Fisher Scientific Inc., Waltham, MA, USA) or the broccoli extract. The cells that were not activated with LPS for 24 h served as the control group. A total of 100 μL of cell culture medium was added to a 96-well plate and a similar volume of Griess reagent was added. The mixture was allowed to react at room temperature for 10 min, and the absorbance was measured at a wavelength of 550 nm. NaNO_2_ (Sigma Chemical Inc., St. Louis, Louis, MO, USA) was used as a standard to plot a standard curve, and the inhibitory effect was expressed as a percentage.

### 2.8. Measurement of PGE_2_ Content

Cells were seeded in a 24-well plate at 1 × 10^5^ (cells·mL^−1^) cells per well, and allowed to grow for 24 h. The medium was changed and LPS and the broccoli extract (5, 10, 20, 40, and 60 ppm) were added and allowed to react for a further 24 h. The Prostaglandin E_2_ ELISA Kit (Cayman Co., Ann Arbor, MI, USA) commercial kit was used to determine the PGE_2_ content of the RAW264.7 macrophage supernatants, as per the manufacturer’s instructions.

### 2.9. Expression Levels of iNOS and COX-2

#### 2.9.1. Total Cellular RNA Extraction

RAW264.7 macrophages were seeded onto a 24-well plate and cultured for 24 h. The medium was removed, and the samples were treated with different concentrations (5, 10, 20, 40, and 60 ppm) of broccoli extract for one day, depending on the experimental treatment, and washed twice with 1 × PBS. An appropriate amount of Trypsin-EDTA was added to detach the cells, and the cells were collected in a microcentrifuge tube and centrifuged at 500× *g* for 5 min. The supernatant was removed, and 1-thioglycerol/homogenization solution and lysis buffer were sequentially added to the cell suspension as per the instructions of the Maxwell^®^ RSC simplyRNA Cells Kit (Promega Co., Madison, WI, USA). Total RNA was extracted using the Maxwell^®^ RSC Instrument.

#### 2.9.2. RT-PCR Analysis

Extracted RNA was reverse transcribed into cDNA using GoScriptTM Reverse Transcription Mix (Promega Co.,, Madison, WI, USA) and oligo (dT) primers, and then PCR was performed using GoTaq^®^ Green Master Mix (Promega Co., Madison, WI, USA). The resulting product was subjected to gel electrophoresis in 1% agarose (Amresco Inc., Solon Ind. Pkwy., Solon, OH, USA) and 0.003% HealthyView nucleic acid stain (Genomics, Taipei, Taiwan) for analyzing the size of the specific fragments, and GeneTools 4.3.7 software was used to quantify and compare the fragments in the gel. The following primers were used: iNOS forward, 5′-AAT GGC AAC ATC AGG TCG GCC ATC ACT-3′, reverse, 5′-GCT GTG TGT CAC AGA AGT CTC GAA CTC-3′; COX-2 forward, 5′-GGA GAG ACT ATC AAG ATA GT-3′, reverse, 5′-ATG GTC AGT AGA CTT TTA CA-3′; β-actin forward, 5′-TCA TGA AGT GTG ACG TTG ACA TCC GT-3′, reverse, 5′-CCT AGA AGC ATT TGC GGT GCA CGA TG-3′ (Mission biotech, Taipei, Taiwan).

#### 2.9.3. GSH/GSSG Ratio

The GSH/GSSG-Glo™ assay (Promega Co., Madison, WI, USA) was used to measure the ratio. RAW264.7 cells were cultured in a 96-well plate containing a medium. After 3 h, the old medium was removed, and either DMEM (control group), 2 μg·mL^−1^ LPS (positive control group), or broccoli extract with different concentrations of SFN were added, and the cells were cultured for 20 h. Total glutathione reagent (50 μL·well^−1^) or oxidized glutathione reagent (50 μL·well^−1^) was added to each well and shaken for 5 min. In addition, the glutathione standard was diluted into 8 different concentrations by a 2-fold serial dilution method, and the total glutathione reagent (50 μL·well^−1^) was added. Luciferin generation reagent (50 μL·well^−1^) was added to each treatment group and the standard group and mixed well and incubated at room temperature for 30 min. Next, the luciferin detection reagent (100 μL·well^−1^) was added and allowed to stand for 15 min, and the luminescence was measured (integration time = 0.3 s). The GSH/GSSG ratio is calculated as follows: ratio GSH/GSSG treated = (μM total glutathione treated – (μM GSSG treated × 2))/μM GSSG treated.

#### 2.9.4. Statistical Analyses

XLSTAT statistical software was used for analysis of variance (ANOVA). Differences in the means between the groups of data were analyzed using Tukey’s test (Tukey’s Honestly Significant Difference Test, Tukey’s HSD). The significance level was kept at *p* < 0.05. Statistical results are expressed in lowercase English letters. Two sets of data marked with completely different letters indicate a statistically significant difference; duplicate or identical letters indicate a lack of a statistically significant difference between the data.

## 3. Results and Discussion

**Figure 1:**
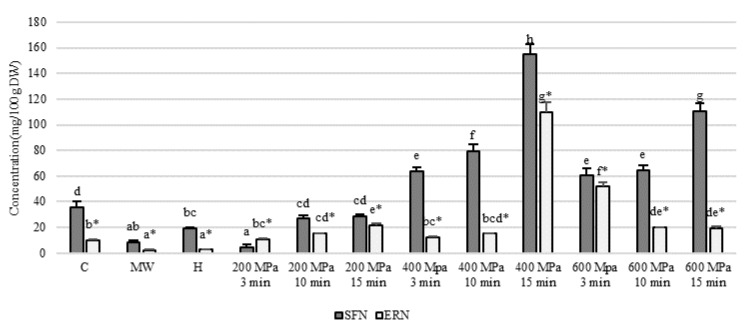
The sulforaphane (SFN) and erucin (ERN) contents of broccoli under different conditions. C: control (untreated); MW: microwave treatment (800 W, 3 min); H: hot water treatment (100 °C, 3 min). The same symbols, noted as superscripts after the letter, means that the ANOVA performed is in the same group. Bars carrying different letters are statistically different at *p* < 0.05.

The calibration curves of sulforaphane (SFN) and erucin (ERN) were established by HPLC analysis. The R2 value of SFN and ERN was 0.9994 and 0.9998, respectively, and the elution time was 16 min and 35 min, respectively. Figure 1 displays the SFN and ERN metabolite content of broccoli treated under different conditions. In the untreated broccoli samples, the SFN content was measured to be 35.59 mg·100 g^−1^, and the ERN content was 10.30 ± 0.21 mg·100 g^−1^. After microwave and hot water treatment, the content is significantly reduced by at least 70%. However, in the high-pressure treatment group, the SFN content increased significantly when the pressure range was kept between 200 and 400 MPa and increased with increasing pressures and treatment times. The highest content was achieved in the group that underwent treatment at 400 MPa for 15 min, obtaining the highest SFN content of 154.79 ± 7.64 mg·100 g^−1^. When the pressure was increased to 600 MPa, the SFN content decreased. Similarly, the highest ERN content was achieved in the group with treatment at 400 MPa for 15 min, obtaining the highest ERN content of 109.86 ± 7.45 mg·100 g^−1^. When the pressure was increased to 600 MPa, there was a significant decrease in ERN content as the treatment time was increased.

Both SFN and ERN are isothiocyanates that are primarily produced by the glucosinolates–myrosinase system. According to the literature, hot water soaks into cruciferous crops during the cooking process, resulting in a 90% loss in glucosinolate and isothiocyanate content. Microwave treatment also destroys myrosinase activity, which prevents glucosinolates from being converted into isothiocyanate and lowers their content [28,29]. In the present study, broccoli was treated with a non-thermal, high hydrostatic pressure of 400 MPa for 15 min. The contents of both the isothiocyanates, SFN and ERN, in the broccoli were significantly increased, but their content decreased when the pressure reached 600 MPa. A study by Westphal et al. (2017) [5] indicated that an increase in pressure can promote the disintegration of plant cell structures, releasing large quantities of myrosinase, which then interacts with the glucosinolates to increase the isothiocyanate content. A study by Eylen et al. (2009) [17] showed that myrosinase enzyme begins to inactivate with increasing pressures. The rate of myrosinase inactivation increases when the pressure is increased over 500 MPa, which reduces the amount of isothiocyanate produced.

All data are presented as the mean ± SD (*n* = 3). The same symbols, noted as a superscript after the letter, means that the ANOVA performed is in the same group. Bars carrying different letters are statistically different (*p* < 0.05).

**Figure 2:**
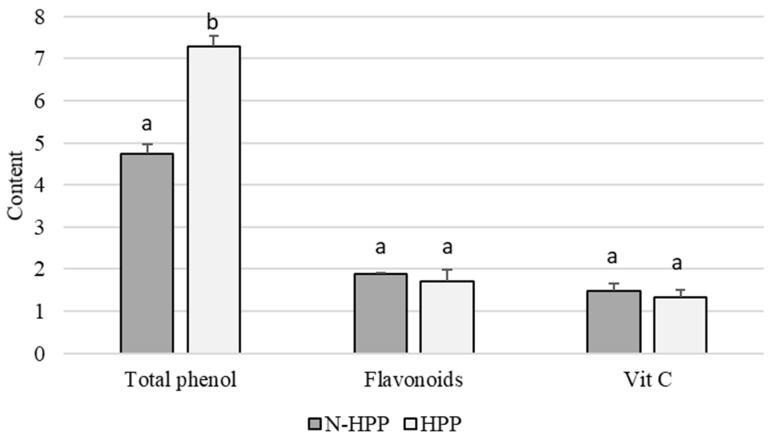
Content of the functional components in the untreated group (N-HPP) and high-pressure group. (HPP-400 MPa, 15 min). Total phenol: GAE mg·g^−1^. Flavonoids: QuE mg·g^−1^. Vit C: mg·g^−1^. All data are presented as the mean ± SD (*n* = 3). Bars carrying different letters on the same parameter are statistically different (*p* < 0.05).

Figure 2 indicates the changes in the functional components of broccoli after high-pressure treatment. TPC is the most significant component before and after high-pressure treatment. The initial TPC of broccoli was 4.73 ± 0.23 GAE mg·g^−1^. After high-pressure treatment, the content increased to 7.28 ± 0.26 GAE mg·g^−1^, an increase of about 1.5-fold. The content of flavonoids and vitamin C were not significantly altered after high-pressure treatment, indicating that high-pressure processing can result in better nutrient retention in foods. Vinicio et al. (2017) [30] reviewed numerous kinetic studies reporting the HPP effects of phytochemicals focused on microstructural changes and found the effects of HPP on the concentration of phenolic compounds are not clear and may either increase, decrease, or not be affected by HPP. This might be due to the large and complex phenolics family that exists in many forms in plants, some found as soluble conjugated glycosides, and some found as insoluble forms typically bound to structural components of the polysaccharides or proteins of the cell wall [31,32]. Different forms of phenolic compounds may react differently under high-pressure treatments. Disruption of the cellular structure causes the compartments to release their contents, and the disassociation of the phenolic compounds from the bound polysaccharides or proteins is generally hypothesized to play a major role. However, the mechanisms by which phytochemicals are released from plant cells remain mostly unknown [30]. Liu et al. (2020) [33] reviewed the current state of knowledge on the internal factors that influence cell wall polysaccharides and polyphenol interactions. In that article, many advanced instrumental analysis methods (ITC, TSC, DLS, NTA, NIR, NMR, CLSM, etc.) were also introduced for the discovery of the exact interaction mechanism, through studies of their morphology, chemical composition, and molecular architecture.

Hence, the content of flavonoids and vitamin C were not significantly altered by the high-pressure treatment of broccoli, whereas TPC increased, indicating that a high hydrostatic pressure is effective in maintaining the heat-sensitive components. Comparisons based on the contents of vitamin C and isothiocyanates showed that these two functional components exhibited different tendencies, suggesting that the increase in isothiocyanate content could be unrelated to vitamin C levels. The results of the present study are similar to those of a study by Prasad et al. (2009) [34], in which high pressures were used to extract these components from longan peels. Since high-pressure treatments can disrupt the hydrophobic bonds in the cellular walls and cell membranes, thereby increasing the rates of substance transfer and facilitating the penetration of solvents into cells, this leads to an increase in the phenolic content. Rodríguez-Roque et al. (2015) [17] and Landl et al. (2010) [35] indicated that vitamin C is a substance that is sensitive to environmental changes, and its stability is easily affected by the presence of oxygen, heat, and heavy metals. High-pressure treatment not only involves no heat, but also inhibits the activity of oxidases, greatly reducing the loss of vitamin C. In addition, Tola and Ramaswamy (2015) [36] also indicated that high-pressure processing does not destroy the covalent, hydrophobic, or ionic bonds present in small molecule components, resulting in better nutrient retention in food.

With respect to the analysis of enzymatic activities, the activities of the MYR, APX, POD, and PPO enzymes were analyzed, and the results are indicated in Table 1. The activity of MYR after high-pressure treatment was significantly higher than in the untreated group, regardless of the blanching process, indicating that high-pressure processing can effectively increase MYR activity. As a comparison with isothiocyanate content, these two values exhibited a similar tendency of change, suggesting that high-pressure stimulation of MYR activity increases isothiocyanate content. There was no significant difference in APX activity after blanching, but after high-pressure treatment, the APX activity exhibited an increasing tendency. This was not consistent with the values obtained for the vitamin C content, suggesting that the increase in isothiocyanate content might not be related to changes in APX activity. The activities of PPO and POD were significantly reduced after blanching and high-pressure treatment, indicating that both processing methods could inhibit oxidase effectively and preserve food quality.

The activity of the enzyme myrosinase after high-pressure treatment was significantly higher than in the untreated group, and this increase in activity exhibited a tendency similar to the change in isothiocyanate content, indicating that high-pressure processing can promote myrosinase activation and thereby increase isothiocyanate production. Wang et al. (2016) [37] and Okunade et al. (2015) [38] applied high-pressure treatment to measure the myrosinase activity in Brussels sprouts and mustards, and their results were similar to that of the present study. Furthermore, Wang et al. (2016) [37] indicated that myrosinase activity is altered based on the environmental pH and that high-pressure treatment affects the ionic balance in food, suggesting that environmental pH is more suitable for myrosinase survival and thus improves the overall activity. The effects of high-pressure processing on the enzymes’ activities are complex and depends a lot on the matrix composition of the tested samples. Wang et al. (2018) [39] studied the high-pressure effects on myrosinase activity and glucosinolate preservation in seedlings of Brussels sprouts and proposed that the effect depends on myrosinase activity and cell permeabilization. The measurable increase of myrosinase activity and content of isothiocyanates can be explained by the interplay of the increased contact between the glucosinolates and myrosinase via membrane permeabilization, induced by the high pressure and availability of myrosinase.

When compared to other enzymes, there were no significant differences in the APX activity levels before and after blanching. This result is in accordance with a previous study by Vicente et al. (2006) [40] in heat-treated strawberries. Their study indicated that heat and oxidative stress induced an increased expression of the gene *apx1* upstream of APX, causing the activity of APX to be retained after heat treatment. APX activity was significantly increased after high-pressure treatment. This was not consistent with the vitamin C content changes, suggesting that an increase in isothiocyanate content might not be related to changes in APX activity. Although the APX enzyme plays an important role in vitamin C metabolism, the measurable content of vitamin C in HPP processed fruits and vegetables is variable due to many possible mechanisms, such as the enhanced extraction of bioactive compounds and the cells’ rupture that releases their cytosol content, caused by the compression effect of the high pressure [41].

POD and PPO are oxidases commonly found in plant cells that primarily use phenolic compounds as substrates. When these two enzymes interact with phenolic compounds, brown colored substances are formed, causing enzymatic browning of plants. In addition, peroxidases are known to oxidize lipids, causing unpleasant odors that affect food quality [42]. After high-pressure treatment of broccoli, the POD and PPO activities were significantly reduced by 51% and 39%, respectively. According to previous studies by Denoya et al. (2015) [43] and Fang et al. (2008) [44], high-pressure techniques can reduce the activity of these enzymes by altering the structures of these proteins, and hence the activities of POD and PPO are significantly reduced after high-pressure processing.

**Figure 3:**
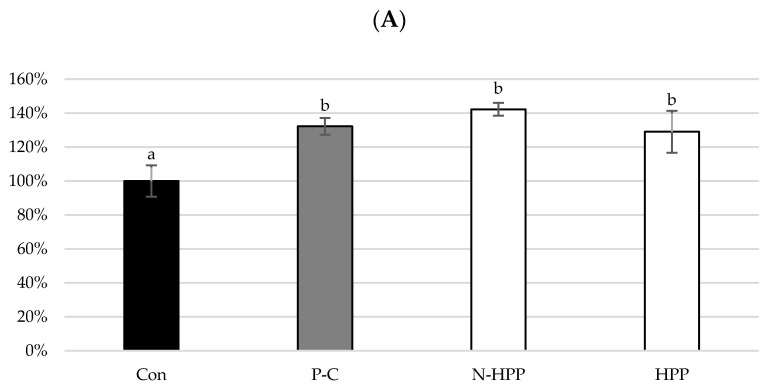

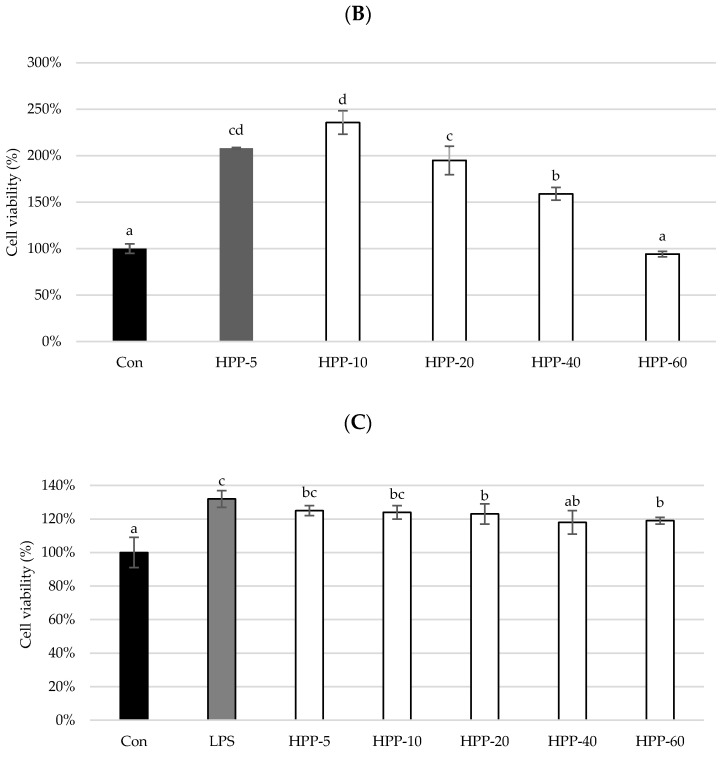
Cell survival rate. (**A**) Effects of the untreated group and high-pressure treated group. ▓Con: control (not LPS induced); ▓P-C: positive control (LPS induced); □N-HPP: LPS induction + Non HPP untreated group; □HPP: LPS induction + high-pressure group, 400 MPa, 15 min. (**B**) Effects of the broccoli extracts of different SFN concentrations without LPS induction. ▓Con: Control; □HPP-5 (not LPS induced + 5 ppm broccoli extract). (**C**) Effects of the broccoli extracts of different SFN concentrations. ▓Con: control (not LPS induced); ▓LPS:(LPS induced); □HPP-5 (LPS induced +5 ppm broccoli extract). All data are presented as the mean ± SD (*n* = 3). Different letters in the same row indicate significantly different results (*p* < 0.05).

Figure 3 shows the effect of the broccoli extract on the viability of RAW264.7 macrophages. The control group was not induced by LPS or the given sample treatment, so its survival rate was 100%. The LPS induction and sample treatment resulted in a higher cell viability compared to the control group (Figure 3A). When different concentrations of SFN broccoli extract were added to the cells without the effect of LPS, cell viability after treatment at every concentration was significantly higher than that of the control group (Figure 3B). With LPS induction, cell viability was not reduced by the increased extract concentration and was significantly higher than that of the control group (Figure 3C).

LPS is a polysaccharide present in the cell wall of Gram-negative bacteria. Its structure contains lipid A, which is a source of endotoxin and can activate macrophages and cause inflammation [45]. After LPS-induced inflammation, the cell viability was not decreased below that of the control group. According to Brandenburg et al. (2010) [46], exposure to low concentrations of LPS did not lead to cell death, but instead stimulated cell viability, so the cell viability of the positive control group was higher than that of the control group. When the broccoli extract was added to the cells, the viability of the treatment group was higher than that of the control group regardless of whether inflammation was induced, indicating that broccoli extract is not toxic to cells. This result is consistent with those of a study by Hwang and Lim (2014) [12] in which different SFN concentrations (7.8–1000 mg·mL^−1^) of the broccoli extract were added to RAW264.7 inflammatory cells. In addition, Guerrero-Beltrán et al. (2010) [47] indicated that SFN could enhance cytoprotection by inducing nuclear translocation of the Nrf2 protein, thereby improving viability.

**Figure 4:**
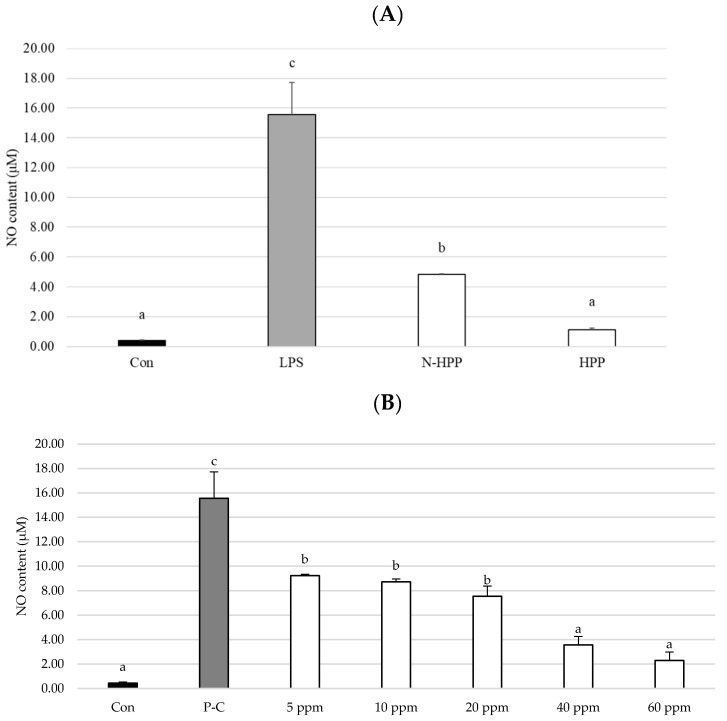
Nitric oxide production. (**A**) Untreated group and high-pressure treated group. ▓Con: control (not LPS induced); ▓LPS: (LPS induced); □N-HPP: LPS induction + Non HPP untreated group; □HPP: LPS induction + high-pressure group, 400 MPa, 15 min. (**B**) Effects of the broccoli extracts of different concentrations. ▓Con: control (not LPS induced); ▓P-C: positive control (LPS induced); □HPP-5 (LPS induced + 5 ppm broccoli extract). All data are presented as the mean ± SD (*n* = 3). Different letters in the same row indicate significantly different results (*p* < 0.05).

The effect of broccoli extract addition to RAW264.7 inflammatory macrophages, and specifically its effects on nitric oxide production, are shown in Figure 4. After the inflammatory cells were treated with broccoli extract, the amount of NO produced was significantly lower than in the positive control group, indicating that the extract has anti-inflammatory potential. After high-pressure treatment, the effect of the extract on inhibiting NO production was improved 1.3-fold compared to the untreated group (Figure 4A). Using SFN as an indicator, the broccoli extract was diluted to 5, 10, 20, 40, and 60 mg.mL^−1^ and added to the inflammatory cells. Figure 4B indicates that the inhibitory effect of the extract is at its best when the concentration of SFN is 60 mg.mL^−1^ and the NO content is reduced by 85% compared to the positive control group.

NO is an important biological indicator in cells that can regulate the physiological processes associated with inflammation [48]. Treatment of cells with LPS causes an inflammatory response, which promotes upregulation of iNOS in macrophages affected by the inflammatory response, leading to a massive increase in the production of NO [49]. In the present study, the NO content in the cells was significantly reduced when treated with broccoli extracts with different SFN concentrations, with higher SFN concentrations yielding better inhibitory effects. The results of the present study are similar to those of Subedi et al. (2019) [50], in which glial cells were treated with purified SFN. This study indicated that SFN can effectively downregulate iNOS expression, thereby decreasing NO production. Yang et al. (2007) [51] applied SFN to retinal microglia in which inflammation was induced by LPS. The NO content decreased significantly when the concentration of SFN was increased between 1.25 and 10 μM. Furthermore, it was observed that the changes in iNOS expression were proportional to the NO content. These studies suggest that SFN primarily reduces iNOS expression and NO production, thereby delaying inflammation.

All data are presented as the mean ± SD (*n* = 3). Different letters in the same row indicate significantly different results (*p* < 0.05).

**Figure 5:**
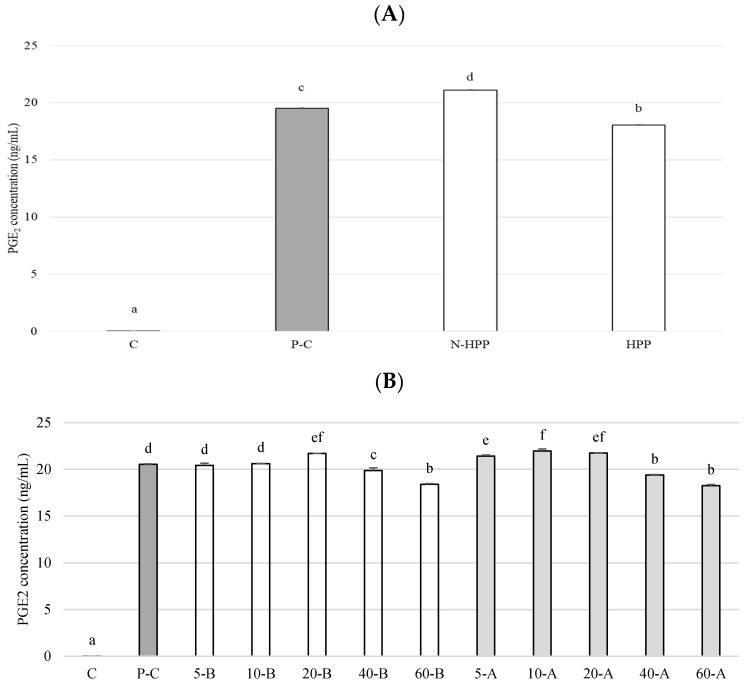
PGE_2_ content. (**A**) Untreated group and high-pressure-treated group. ▓C: control, ▓P-C: positive control (LPS induced); □N-HPP: LPS induction + Non HPP untreated group; □HPP: LPS induction + high-pressure group, 400 MPa, 15 min. (**B**) Broccoli extracts of different concentrations, pre-/post-LPS treated. ▓C: control (not LPS induced); ▓P-C: positive control; □5-B broccoli extracts 5 ppm, pre-LPS treated; ▓5-A broccoli extracts 5 ppm, post-LPS treated. All data are presented as the mean ± SD (*n* = 3). Different letters in the same row indicate significantly different results (*p* < 0.05).

A pre-LPS induction and a post-LPS induction group were used to simulate the treatment and prevention, respectively. Figure 5 shows the effect of the broccoli extract on the prostaglandin E_2_ (PGE_2_) content in RAW264.7 inflammatory macrophages. After LPS induction, the PGE_2_ content of the cells increased significantly, indicating that the cells were in an inflamed state. After the addition of broccoli extract, the high-pressure broccoli extracts inhibited PGE_2_ production compared to the untreated group (Figure 5A). Regardless of the LPS induction strategy done first or later, once the broccoli extract was diluted, the inhibitory effects on PGE_2_ was reduced both before and after LPS induction, and the PGE_2_ content doubled compared to pre-dilution, indicating that the concentration of SFN in the broccoli extract must be higher than 60 mg·mL^−1^ in order for the inhibitory effect to be significant (Figure 5B).

PGE_2_ is one of the most abundant prostaglandins in the human body and is involved in many physiological and pathological processes, including cancer and inflammation [52]. Upon LPS induction, COX is rapidly activated in cells, prompting the conversion of large amounts of arachidonic acid into prostaglandins (PG), including PGI2, PGE2, and other molecules, which lead to inflammation. A large amount of PGE_2_ is present after LPS-induced inflammation. The PGE_2_ content is significantly reduced after addition of high-pressure-treated broccoli extracts, and this effect is greater than in the untreated group. However, when the extract is diluted to different SFN concentrations, the inhibitory effect on PGE_2_ exhibited a decreasing tendency. Park et al. (2019) [53] treated cells with LPS-induced inflammation with broccoli extracts and found that the PGE_2_ content was significantly reduced, which is similar to the findings of the present study. Qi et al. (2016) [54] used LPS to induce lung injury in BALB/c mice that were previously treated with SFN and found that the PGE_2_ content in these mice were significantly reduced, indicating that SFN has the potential to delay inflammation. In addition, these two studies also indicated that the amounts of PGE_2_ produced are correlated with the COX-2 expression levels, suggesting that SFN primarily inhibits PGE_2_ by reducing COX-2 expression.

**Figure 6:**
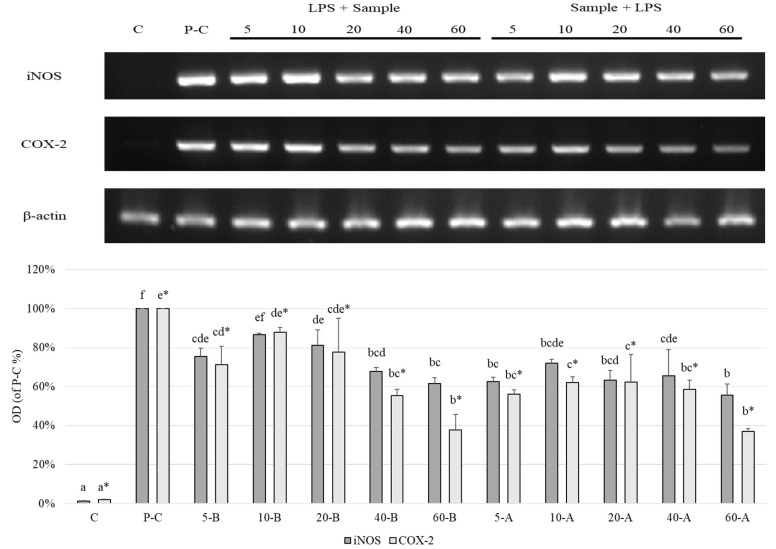
Effects of different extracts of broccoli on the iNOS, COX-2, and β-actin gene expression in RAW264.7 macrophage cells. C: control; P-C: positive control; 5-B: broccoli extracts 5 ppm, pre-LPS treated; 5-A: broccoli extracts 5 ppm, post-LPS treated. All data are presented as the mean ± SD (*n* = 3). The same symbols, noted as superscripts after the letter, means that the ANOVA performed is in the same group. Bars carrying different letters are statistically different at *p* < 0.05.

Figure 6 shows the effect of broccoli extracts on iNOS and COX-2 in inflammatory cells. After the cells were treated with broccoli extracts, the expression level of iNOS was lower than in the positive control group, with the post-induction group, treated with a SFN concentration of 60 mg.mL^−1^, exhibiting the best inhibitory effect on iNOS, around 61%. With regards to COX-2 expression, the changes in expression levels were similar to that in iNOS, and the pre-LPS induction group exhibited a significant concentration-dependent effect, with expression decreasing with increasing SFN concentration. In addition, both induction treatment strategies exhibited the best inhibitory effects on COX-2 gene expression at a concentration of 60 mg.mL^−1^, and the inhibitory effects were measured to be 46% and 35%, respectively.

iNOS is an upstream enzyme that produces nitric oxide, and COX-2 is a pivotal enzyme for the production of PGE_2_. Inflammation stimulates increased expression of both enzymes and promotes production of large amounts of inflammatory cytokines [55]. Many studies have found that excessive expression of iNOS and COX-2 causes massive production of inflammatory factors such as NO and PGE_2_. SFN can effectively inhibit the expression of these two proteins, thereby regulating inflammatory response [56,57,58,59]. In the present study, applying high-pressure-treated broccoli extracts to inflammatory macrophages had the best inhibitory effect on iNOS or COX-2 when the SFN concentration was 60 mg.mL^−1^. Comparisons based on the results of iNOS and COX-2 inhibition with NO production and PGE_2_ content showed that the changes in iNOS and NO production were correlated, which is consistent with the results of the aforementioned studies, but the COX-2 and PGE_2_ content were different. Cells contain two COX isoenzymes, namely, COX-1 and COX-2. COX-1 functions primarily as a housekeeping gene and can stabilize the physiological functions of cells. COX-2 is generally considered to be activated by inflammation. However, studies related to neurodegenerative diseases and neuroinflammation have found that the expression levels of COX-1 is associated with the production of PGE_2_ and inflammatory cytokines in microglia, showing that COX-1 not only stabilizes cell physiology, but also promotes inflammation [60,61]. In addition, Qin et al. (2016) [62] and Zhou et al. (2012) [52] found that the mechanism of SFN inhibition of PGE_2_ content might be achieved through regulating the expression of microsomal prostaglandin E synthase 1 (mPGES-1) downstream of COX-2, rather than by inhibiting COX-2 expression. These studies suggest that, in addition to COX-2, the factors affecting the synthesis of PGE_2_ are also regulated by COX-1 and mPGES-1, resulting in differences in the COX-2 expression levels and PGE_2_ content.

**Figure 7:**
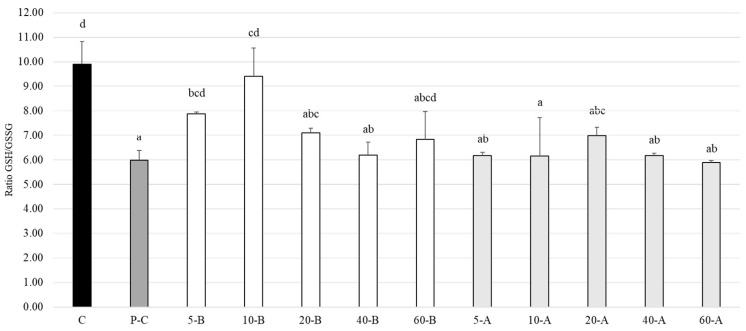
Effects of the broccoli extracts with different SFN concentrations on the ratio of GSH/GSSG in RAW264.7 macrophages. All data are presented as the mean ± SD (*n* = 3). Different letters in the same row indicate significantly different results (*p* < 0.05).

The antioxidant effect of broccoli was evaluated by the ratio of reduced to oxidized glutathione (GSH/GSSG) in cells. Figure 7 shows that the GSH/GSSG ratio of the control group not induced by LPS was 9.90, whereas this ratio (5.99) was significantly reduced in the positive control group treated with only LPS. In the LPS pre-induction group, the ratio was significantly higher than that of the positive control group when the SFN concentration was 5 mg.L^−1^ and 10 mg.L^−1^. The antioxidant effect was best when the SFN concentration was 10 mg.L^−1^, with a GSH/GSSG ratio of 9.41.

Glutathione (GSH) is an important indicator of oxidative/nitrative stress in organisms. It can metabolize ROS and RNS to clear potentially toxic oxidation products and reduce oxidative and nitrative damage in cells. In addition, GSH is also a coenzyme of glutathione peroxidase, which protects the sulfhydryl group of this enzyme from oxidation and preserves its activity [63]. Oxidation of GSH yields glutathione disulfide (GSSG), and the alterations in the ratio between these two are associated with redox balance in cells. Hence, the GSH/GSSG ratio is often used as an indicator to evaluate the degree of cellular oxidation [64]. In our present study, the GSH/GSSG ratio was significantly reduced, which is consistent with the results of a previous study by Yamada et al. (2006) [65] regarding *n* dendritic cells induced with LPS. The literature indicates that ROS is generated in large quantities when cells are in an inflamed state, which reduces the GSH/GSSG ratio. When treated with broccoli extracts with different SFN concentrations, the antioxidant effect is best at an SFN concentration of 5 mg.L^−1^ or 10 mg.L^−1^, which is consistent with the results of studies by Kim et al. (2003) [66] and Heiss et al. (2001) [67], who respectively used HepG2-C8 cells and RAW264.7 macrophages for analyzing oxidative stress. After treatment with low concentrations of SFN, the GSH content in the cells increased with increasing time in culture, and the antioxidant capacity of the cells was significantly improved.

## 4. Conclusions

This study demonstrated that high-pressure treatment could effectively increase the isothiocyanate content in broccoli. Specifically, the best results were achieved with processing conditions of 400 MPa for 15 min. The mechanism for this change is primarily due to the high-pressure processing strategy, which stimulates myrosinase activity, thereby increasing the efficiency of the glucosinolate hydrolysis and increasing the isothiocyanate content. With regards to the functional components, high-pressure processing did not affect the vitamin C or flavonoid content in broccoli, and increased the TPC. When compared to traditional thermal processing, high-pressure processing can prevent the loss of heat-sensitive components. In addition, the activity of PPO and POD in broccoli tended to decrease after being subjected to high-pressure treatment, indicating that this processing technique can help inhibit oxidase activity and maintain food quality. With respect to cellular experiments, the varying concentrations of the broccoli extracts applied to the cells did not affect the viability of the RAW264.7 macrophages. Further evaluation of its anti-inflammatory and antioxidant effects showed that broccoli extracts could effectively inhibit NO production, PGE_2_ content, and iNOS and COX-2 protein expression levels. At low concentrations, SFN significantly increased the GSH content and reduced GSSG production, indicating that broccoli has the potential to delay inflammation and reduce oxidative stress. In conclusion, high-pressure processing of broccoli not only adds value to fresh food and provides increased nutritional value, but also allows it to be used as a raw material or additive for the development of healthy foods, in so doing maximizing the utilization value of broccoli.

## Figures and Tables

**Table 1:** Changes in enzyme activity in broccoli before and after high-pressure treatment.

	Non-Blanched		Blanched	
	Non Processed	HPP Processed	Non Processed	HPP Processed
MYR (μmol·g^−1^·min^−1^)	165.75 ± 3.75 ^b^	267.25 ± 28.75 ^c^	123.50 ± 2.00 ^a^	250.75 ± 15.75 ^c^
APX (mM·min^−1^·mg^−1^ protein)	0.142 ± 0.002 ^a^	0.324 ± 0.004 ^b^	0.112 ± 0.011 ^a^	0.361 ± 0.026 ^b^
PPO (∆A·min^−1^)	0.0067 ± 0.0003 ^d^	0.0049 ± 0.000 ^c^	0.0041 ± 0.0001 ^b^	0.0025 ± 0.0001 ^a^
POD (∆A·min^−1^)	0.161 ± 0.007 ^d^	0.148 ± 0.015 ^c^	0.043 ± 0.002 ^b^	0.021 ± 0.004 ^a^

All data are presented as the mean ± SD (*n* = 3). Different letters in the same row indicate significantly different results (*p* < 0.05). MYR: myrosinase; APX: ascorbate peroxidase; PPO: polyphenol oxidase; POD: peroxidase. The activity of the PPO and POD is expressed in the units of absorbance change per min (∆A·min^−1^).
